# miRNA Expression Profiles in Luminal A Breast Cancer—Implications in Biology, Prognosis, and Prediction of Response to Hormonal Treatment

**DOI:** 10.3390/ijms21207691

**Published:** 2020-10-17

**Authors:** Erik Kudela, Marek Samec, Lenka Koklesova, Alena Liskova, Peter Kubatka, Erik Kozubik, Tomas Rokos, Terezia Pribulova, Eva Gabonova, Marek Smolar, Kamil Biringer

**Affiliations:** 1 Department of Obstetrics and Gynecology, Martin University Hospital and Jessenius Faculty of Medicine in Martin, Comenius University of Bratislava, 03601 Martin, Slovakia; marek.samec@uniba.sk (M.S.); koklesova.lenka@gmail.com (L.K.); liskova80@uniba.sk (A.L.); erik.kozubik@gmail.com (E.K.); rokos1@uniba.sk (T.R.); pribulova.terezia@gmail.com (T.P.); kamil.birnger@uniba.sk (K.B.); 2 Department of Medical Biology, Jessenius Faculty of Medicine, Comenius University in Bratislava, 03601 Martin, Slovakia; peter.kubatka@uniba.sk; 3 Clinic of Surgery and Transplant Center, Jessenius Faculty of Medicine in Martin, Comenius University in Bratislava, 03601 Martin, Slovakia; egabonova@gmail.com (E.G.); marino.smolar@gmail.com (M.S.)

**Keywords:** breast cancer, luminal A, estrogen receptor, miRNA, tamoxifen, prognosis

## Abstract

Breast cancer, which is the most common malignancy in women, does not form a uniform nosological unit but represents a group of malignant diseases with specific clinical, histopathological, and molecular characteristics. The increasing knowledge of the complex pathophysiological web of processes connected with breast cancercarcinogenesis allows the development of predictive and prognostic gene expressionand molecular classification systems with improved risk assessment, which could be used for individualized treatment. In our review article, we present the up-to-date knowledge about the role of miRNAs and their prognostic and predictive value in luminal A breast cancer. Indeed, an altered expression profile of miRNAs can distinguish not only between cancer and healthy samples, but they can classify specific molecular subtypes of breast cancer including HER2, Luminal A, Luminal B, and TNBC. Early identification and classification of breast cancer subtypes using miRNA expression profilescharacterize a promising approach in the field of personalized medicine. A detection of sensitive and specific biomarkers to distinguish between healthy and early breast cancer patients can be achieved by an evaluation of the different expression of several miRNAs. Consequently, miRNAs represent a potential as good diagnostic, prognostic, predictive, and therapeutic biomarkers for patients with luminal A in the early stage of BC.

## 1. Introduction

Despite multiple information campaigns and screening programs, the incidence of breast cancer (BC) is extremely high worldwide. It is the most common malignancy in women, except in the East African region, where cervical cancer is on the top [1]. According to the GLOBOCAN 2018 database, 2 million new cases of BC were diagnosed worldwide in 2018, and 626,000 people died, which is the fourth highest mortality after lung, stomach, and liver cancer [2].

BC does not form a uniform nosological unit but represents a group of malignant diseases with specific clinical, histopathological, and molecular characteristics. Morphological classification based on tumor size and grading has long been shown to be insufficient. Currently, molecular analytical methods help us determine the prognostic and predictive factors of cancer. Perou and Sorlie first proposed the terminology of “molecular classification” in BC with a comprehensive study demonstrating differences in gene expression [3]. Routine assessment of tumor immunophenotype includes examination for estrogen receptors (ERs), progesterone receptors (PRs), and human epidermal growth factor receptor 2 (HER2) expression. These are prognostic markers and important predictive factors for hormonal and anti-HER2 targeting therapy. 

ER and PR are hormone receptors that stimulate the growth of both normal and neoplastic breasts. Their expression is present in approximately 75% of all BC. ER/PR-positive tumors are usually low-grade and less aggressive [4]. A small percentage of tumors show only one hormone receptor positivity. These tumors are more aggressive and less sensitive to hormonal therapy compared to ER/PR-positive tumors [5]. Based on the presence of the expression of these receptors examined by immunohistochemistry, BC can be divided into four basic subtypes that correlate with mRNA intrinsic subtypes-Luminal A (ER+, PR≥20%, HER2−, Ki67 < 20%), Luminal B (ER+, PR < 20% and/or HER2+ and/or Ki67≥ 20%), HER2 subtype (ER−, PR−, HER2+), basal-like (triple-negative: ER−, PR−, HER2−) [6]. Some authors report a fifth group of tumors called normal-like (ER+, PR+/−, HER2−, Ki67 low). It is a tumor that, in the early stages, expresses genes like normal breast epithelium. However, it is a controversial group that was later described as artificial and is not widely used in common practice [7].

The latest generations of anti-cancer drugs deserve even more detailed molecular stratification that would help also understand the tumor evolution and resistance to therapy. Even in intrinsic molecular subtypes we see substantial variations in tumor biology with basal-like tumors having the greatest diversity [8]. The novel classification based on the somatic copy number alterations stratifies breast tumors into ten integrative cluster subtypes associated with distinct clinical outcome and response to therapy. Six of these integrative cluster groups are represented by ER dominance compared to two groups of intrinsic types with ER positivity (luminal A and B) [9]. A taxonomy should be dynamic, copying the newest research, knowledge, diagnostic and therapeutic modalities. In our article we present the up-to-date knowledge about the role of miRNAs, their prognostic and predictive value in luminal A BC (breast cancer).

## 2. DifferentialMicroRNA Expression in Luminal BCSubtypes

About 21,000 protein-coding genes thathas been characterized in human genome represent less than 2 % of total genome. The rest but the vast majority of human transcriptome is represented by non-coding RNAs consisting of small RNAs, long non-coding RNAs (lncRNAs) and pseudogenes [10]. These RNA transcripts, especially competing endogenous RNAs (ceRNAs) regulate each other at post-transcription level by competing for shared miRNAs. CeRNA networks describe the interplay between the function of protein-coding mRNAs with non-coding RNAs such as miRNA, lncRNA, pseudogenic RNA, and circular RNA [11]. Small non-coding RNAs, including transfer RNAs, miRNAs and small-interfering RNAs, small nuclear RNAs, small nucleolar RNAs, PIWI-interacting RNAs, and transcription initiation RNAs, are associated with different specific function in translation of mRNAs, post-translational RNA silencing, splicing, ribosomal RNA modification, transposon repression, and transcription regulation, respectively [12]. On the other hand, lncRNAs are implicated in various biological processes (from pluripotency to immune response, RNA gene *XIST* and the role in dosage compensation) but a large number of lncRNAs are not functionally characterized.

**Figure 1:**
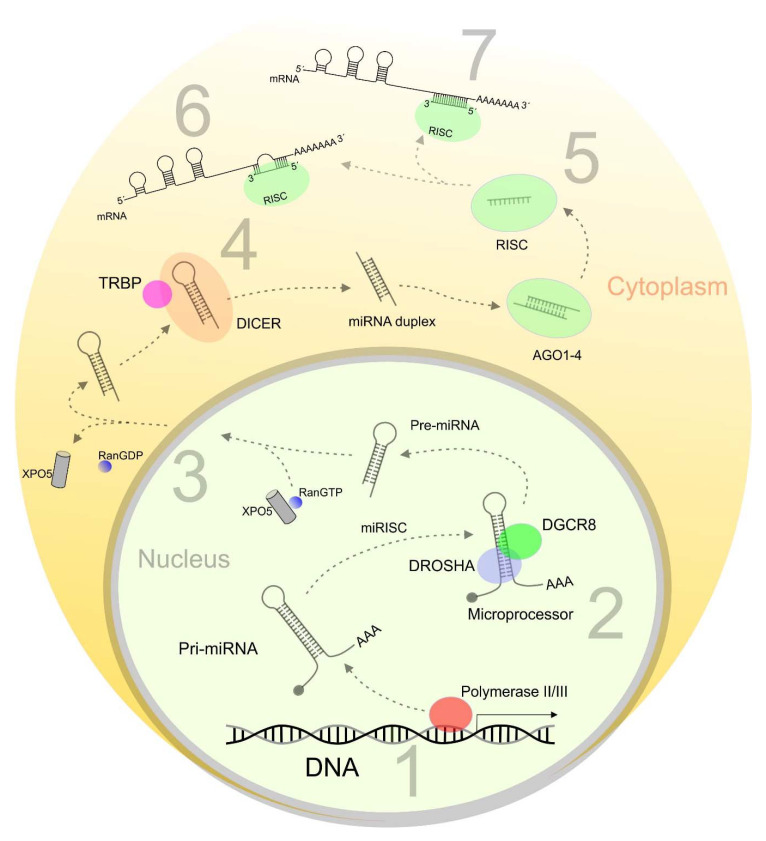
MicroRNA biogenesis. **1**: Transcription; **2**: Drosha processing; **3**: Nuclear export by Exportin-5; **4**: Dicer processing; **5**: Mature miRNA loaded into RISC; **6**: Inhibition of translation; **7**: Degradation of targeted mRNA. MicroRNA is transcribed by polymerase II/III as primary miRNA (Pri-miRNA). In the next step, pri-miRNA is cleavaged by the microprocessor (DGCR8 and DROSHA) to generate precursor miRNA (Pre-miRNA) 70nt length. Pre-miRNA is further exported into the cytoplasm by exportin5 (XPO5) and RanGTP. Pre-miRNA is processed into mature miRNA by DICER. One strand of mature miRNA is loaded into the miRNA-induced silencing complex (miRISC). MiRISC consists of Argonaut proteins (AGO) and DICER. MicroRNA in complex with miRISC has effector function by sequence complementarity leading to suppression of translation or degradation of targeted mRNAby binding to the 3′-untranslated regions of mRNA.Pri-miRNA, primary miRNA; DGCR8, DiGeorge syndrome critical region 8; Pre-miRNA, precursor miRNA; XPO5, exportin 5; AGO, argonaut; miRISC, miRNA-induced silencing complex; TRBP, TAR RNA binding protein.

Among the all small non-coding RNAs, microRNAs represent a group of evolutionary conserved non-coding RNAs that regulate gene expression via translational repression or mRNA degradation [13]. Since their discovery in 1993, miRNAs are still a growing field of cancer-associated research [14]. The biogenesis of miRNA is a multi-step process regulated by specific enzymes and proteins contributing to miRNA processing (Figure 1) [15]. Single miRNA can target multiple genes anda group of different miRNAs may regulate the same gene. This fact determines the important role of the short RNA sequences in almost all biological processes in the cell [16]. Aberrant expression of miRNAs is directly associated with numerous malignancies, including BC. Indeed, an altered expression profile of miRNAs can distinguish not only between cancer and healthy samples, but they can classify specific molecular subtypes of BC including HER2, Luminal A (LumA), Luminal B (LumB), and TNBC [6,17]. Early identification and classification of BC subtypes using miRNA expression profiles characterizea promising approach in the field of personalized medicine [15].

As was mentioned above, LumA represents a molecular subtype with a better prognosis compared to TNBC or HER2 [18]. Immunohistochemical (IHC) characterization of individual molecular subtypesaccording to IHC markersis a fundamental technique for the classification of BC [19,20,21]. The progress in molecular biology aimed at miRNA expression profiles demonstrates a prospective way to improve BC classification, including better determination of LumA phenotype. Recently, several studies focus on the analysis of differentiation between BC subtypes and specific miRNAs profiles [22,23,24,25,26,27]. Søkilde et al. investigated intrinsic subtypes using miRNA expression profiles in 186 BC cases. They identified an alteration in miR-99a/let-7c/miR-125b miRNA cluster associated with proliferative signaling including JAK, STAT3, c-Myc, RAS, AKT/mTOR or ETS1. Acquired data indicated an increased level of the analyzed miRNAs in LumA patients compared to LumB [28]. Interestingly, the level of miR-152-3p, which serve as a tumor suppressor regulating BC cells proliferation via PIK3CA, was lower in patients with LumA than in patients with LumB in the study evaluating miRNA expression profile in blood obtained from 106 patients with newly diagnosed BC [29]. Furthermore, an increased level of miR-29c-5p and miR-30a-3p and decreased level of miR-130b-3p, miR-185-5p, miR-362-5p, and 378a-3p were observed in patients with LumA while its clinical value needs to be further evaluated in clinical setting, authors of the study analyzed two datasets consisting of 186 healthy tissues, 18 ductal carcinomas in situ, and 1338 invasive breast carcinomas [30]. Moi et al. focused on the alterations between miRNA expression and molecular subtypes in the cohort of Norwegian women. They analyzed FFPE from 102 cancer and 36 benign samples. The data showed a significantly decreased level of miR-17-5p and miR-20a-5p, demonstrated to play an important role in the invasion and migration of cancer cells via Wnt/β-catenin suppression, in the group ofLumA patients [31]. Another study evaluated the relative and absolute expression of certain miRNAs isolated from whole blood samples (*n* = 38). Experimental data from absolute RT-qPCR quantification revealed that a combination of three miRNAs miR-195 (downregulated), miR-145, and miR-486 (upregulated) had the best diagnostic value for patients with LumA. On the other hand, results from relative RT-qPCR quantification detected the upregulation of miR-155 and miR-486 and downregulation of miR-195. Compared to relative quantification, the absolute quantification technique is better to determine the expression level of miRNA isolated from blood (AUC = 0.657 vs AUC = 0.875) [32]. In addition, an expression of miR-1290 was decreased in ER^high^, Ki67^low^tumors in the study evaluating the correlation of miRNA expression profiles and clinicopathological factors (*n* = 64). Interestingly, in silico analysis revealed that predictive targets of miR-1290 include Bcl-2, FOXA1, MAPT, and NAT1 [33]. In a meta-analysis of independent studies, van Schooneveld et al. defined specific miRNAs for each intrinsic subtype of BC. They identified a higher expression of let-7c, let-7f, and miR-10a associated with LumA [34]. In another study, Iorio et al. evaluated miRNAs expression profile specific for the individual BC molecular subtypes. In the LumA subtype, the results showed miR-191 and miR-26 upregulation, while level miR-206 was reduced [35]. Moreover, Blenkiron et al. analyzed levels of 309 miRNAs in 93 BC samples. They detected nine miRNAs including miR-100, -99a, -130a, -126, -136, -146b (upregulated), and miR-15b, -107, and 103 (downregulated), which can distinguish LumA from LumB [36].

All previously described studies show an association between miRNA expression profile and BC intrinsic subtypes. According to the specific molecular signatures, miRNAs can define individual steps of carcinogenesis, metastasis development, or chemo/radioresistance, and thus predict prognosis for patients with LumA and other molecular subtypes of BC.

## 3. Specific miRNA Expressionin Early and Metastatic Stage in LumA BC

BC appears as a local disease, but later stages can lead to the metastasis to lymph nodes and distant organs [37]. It is important to detect the primary tumor early to prevent the spread of cancer to metastasis.An expression of miRNA in different stages of BC is variable mainly due to the association of various potential mechanisms [38]. A detection of sensitive and specific biomarkers to distinguish between healthy and early breast cancer (EBC) patients can be achieved by an evaluation of different expression of several miRNAs. Consequently, miRNAs represents potential as good diagnostic, prognostic, predictive, and therapeutic biomarkers for patients with LumA in the early stage of BC [39].

Several studies focused on the differences in miRNAs expression between patients with LumA and healthy controls. Circulating miR-16, miR-21, miR-155, and miR-195 were increased in the serum levels of patients with EBC compared to healthy controls. The same results were observed in serum of patients with LumA compared to healthy controls. Finally, the measurement of the level of circulating miRNAs by BRCA assay, especially the combination of miR-16, miR-21, miR-155, and miR-195, could be used to detect LumA BC [40]. Moreover, the expression of circulating miR-10b, miR-21, miR-145, miR-155, miR-195 was increased in the blood of predominantly early stage and LumA patients compared to healthy controls but only miR-195 expression was specific to the EBC cohort compared to other cancer types and healthy control. An increased sensitivity in the differentiation between BC patients and controls can be attributed to the combination of circulating miRNAs whose expression can be associated with the correlation with disease burden (miR-195) and promotion of cell migration, invasion, and epithelial-mesenchymal transition (EMT) (miR-155) [41].

The reduced expression of miR-181a and miR-652 and unchanged expression of miR-29a was observed in the blood of women with LumA-like BC compared to healthy controls, irrespective of nodal status or stage of disease suggesting that altered expression of miRNAs have an important biomarker characteristic in both early and late stage disease as well as its important role as potential miRNA-related therapeutic strategies [42]. Similarly, the expression of miR-23a-3p and miR-152-3p (negatively regulating PI3KCA expression inhibiting the suppression of BC cell proliferation) revealed lower levels in the blood of patients with LumA, especially in stage I–II, when compared with healthy controls suggesting the potential in early detection of BC [29].

Several comparative studies evaluated miRNAs expression in various patients with BC (age, surgery) the decreased level of circulating miR-338-3p, miR-223, and miR-148a and higher level of miR-107 was observed in post-operative compared to pre-operative samples from post-menopausal women with a molecular characteristic corresponding to LumA including ER+ HER2− EBC. However, the alteration in expression of these miRNAs in the determination of their potential as clinical biomarkers should be validated in larger prospective studies [43].

Study focusing on LumA in vitro revealed an increased expression level of miR-1273g-3p in MCF-7 BC cells compared to normal Hs 578Bst breast cells. Similar data were observed in breast ductal cancer patients compared with healthy donors. Results revealed the potential of miR-1273g-3p, whose increased expression is associated with BC progression by regulating PTEN, as a biomarker for early breast ductal cancer diagnosis [44]. An evaluation of various miRNA levels is important to distinguish the early stage of LumA subtype of BC from non-cancer disease. Different expression of miRNA between EBC patients and healthy controls or other BC stage was revealed in several studies, which are summarized in Table 1 and suggested to be as potential diagnostic, prognostic, predictive, and therapeutic biomarkers.

Circulating miRNAs can be used as markers to identify metastatic disease as was demonstrated by an evaluation of plasma extracted RNA from BC patients that revealed a significant decrease in the expression of miR-195 and an increase in the expression of miR-331, with their molecular roles related to metastatic processes including proliferation, angiogenesis and EMT, in metastasized LumA patients when compared to patients with local disease or healthy controls [45].

The association between miRNA pattern and the process of metastasis can be clearly demonstrated by the use of human BC cell lines belonging to the LumA molecular subtype including MCF-7 [46] and T47D [47]. The expression of ERα in LumA metastatic lesions is probably a result of the epithelial differentiation of LumA cancer stem cells upon colonizing a site of metastatic spreading or to the reversion of EMT-generated ERα-negative metastatic cells back to ERα-positive state during the reverse process of EMT also known as mesenchymal–epithelial transition (MET). Anyway, a relationship between miRNAs targeting ERα has also been examined [48]. ERα was identified as a direct target of miR-203 in MCF-7 cells while the upregulation of miR-203 inhibited estradiol-induced increase in viability, migration and invasion and decreased the protein expression of ERα in MCF-7 cell line [49].

Anoikis is a phenomenon describing apoptotic cell death as a consequence of insufficient cell-matrix interactions [50] and preventing cancer cells from surviving the detachment from the primary tumor site. Nevertheless, in order to become more aggressive and metastatic, cancer cells can develop a resistance to anoikis and undergo changes including EMT [51]. The resistance to anoikis is therefore a hallmark of metastatic cancer cells. MiR-6744-5p was identified to be downregulated in anoikis-resistant sub-cell line (MCF-7-AR6) generated from MCF-7 cells. Moreover, MCF-7-AR6 was also associated with increased migration when compared with MCF-7. However, the overexpression of miR-6744-5p increased while its knockdown decreased the anoikis sensitivity in the MCF-7. Additionally, the overexpression of miR-6744-5p was related to the increased expression of E-cadherin [50], an adhesion molecule whose downregulation facilitates the tumor invasion and metastasis and contributes to the phenotypic appearance of EMT [52]. Furthermore, a xenobiotic metabolizing enzyme—*N*-acetyltransferase 1 (NAT1)—was identified as a direct target of miR-6744-5p. The processes by which NAT1 could contribute to anoikis resistance are not fully clarified yet; however, the possible mechanisms include the DNA damage through chemical carcinogenesis or inhibition of reactive oxygen species to suppress anoikis [50]. 

Moreover, the attenuation of proliferative, migratory and invasive properties of MCF-7 cells were attributed to the overexpression of miR-765 [53]. In addition, the overexpression of miR-628 increased the migration and invasion of BC stem cells (BCSCs) in MCF-7 via downregulation of the vimentin and Snail expression and upregulation of the E-cadherin expression. miR-628 can potentially suppress the migration and invasion of BCSCs of MCF-7 cells through regulation of its direct target SOS1 [54]. Furthermore, an overexpression of the mitochondrial calcium uniporter (MCU) in MCF-7 cells led to the increase in migration and invasion in vitro and lung metastasis in vivo. Moreover, increased migration and invasion of MCF-7 cells and enhanced glucose uptake was associated with the inhibition of miR-340. Therefore, the ability to regulate BC metastasis via modulation of glycolysis could be attributed to miRNA-340 that targets MCU [55]. Importantly, mammospheres derived from MCF-7 cells represent the model of BCSCs and metastatic tumor tissues with enhanced migration and invasive properties. Importantly, the reduced expression of miR-200c, increased expression of miR-30c as well as increased expression of stem cells markers (*OCT4*, *SOX2*, *c-MYC*) and EMT-related genes (*SNAIL1*, *CDH2*, *TWIST1/2*) was observed in mammospheres, similarly as in case of BC tissues of grade I/II patients [56]. Additionally, a downregulation of miR-145-3p-induced metastasis evaluated in MCF-7 cells in which the hypoxia and serum deprivation was used to mimic *in vivo* cancer microenvironment and trigger metastasis [57]. 

Besides, the overexpression of miR-520c-3p reduced the invasiveness and migration of both MCF-7 and T47D cells demonstrated via an increased level of E-cadherin and decreased level of vimentin and fibronectin [58]. Similarly, miR-206 overexpression suppressed migration, invasion and EMT of MCF-7 and T47D cells via an inhibition of transforming growth factor beta (TGF-β), neuropilin-1 (NRP1), and *SMAD2* [59]. Moreover, the expression of miR-190 was found to be low in BC cell line T47D. Further analysis revealed that overexpression of miR-190 inhibited the process of EMT and angiogenesis [60]. As is shown in Table 2, the evaluation of miRNAs shows a specific pattern of their expression in metastatic BC while the effects of miRNA regulation on processes associated with metastatic spread of LumA BC, such as e.g., anoikis, migration, invasiveness, or EMT, have been demonstrated in in vitro studies conducted on BC cell lines belonging to the LumA molecular subtype.

As was discussed above, the expression of miRNAs has been demonstrated to be different among patients with LumA and other molecular subtypes or healthy controls. The expression of specific miRNAs correlates with BC disease burden and isalso associated with various mechanisms related to BC, such as proliferation, promotion of BC migration, invasiveness, or other processes of BC progression [42,43,48]. Above all, despite that the evaluation of specific miRNA expression represents a potential tool applicable in the differentiation between early and metastatic stage of BC, miRNA expression may be also applicable in the determination of miRNA-regulating therapeutic strategies [42]. The evidence dealing with the evaluation of miRNA expression in the identification of metastatic BC in clinical studies is poor. Nevertheless, the results of preclinical research analyzing the expression of miRNAs in human BC cell lines belonging to LumA molecular subtype revealed that changes in the expression of specific miRNAs arerelated to metastasis-associated mechanisms including changes in migration, invasiveness, EMT or stem cell markers [49,53,55,57,59]. Nevertheless, the usefulness of miRNA expression as clinical biomarker need to be evaluated by larger prospective studies [43].

## 4. miRNAs and BC Prognosis

Traditional prognostic factors in BC patients include number of positive axillary lymph nodes, tumor size, tumor grade, lymphovascular invasion, and the status of hormonal receptors (ER, PR, and HER2). On the other hand, the development of any kind of cancer involves altered regulation of proliferative and growth-inhibitory pathways, activation of oncogenes and inhibition of tumor-suppressor genes. The increasing knowledge of the complex pathophysiological web of processes connected with BC development allows the development of predictive and prognostic gene expression and molecular classification systems with improved risk assessment and individualized treatment.

Epigenetic regulation and its use in prognostic and predictive models are modern era phenomenon. miRNAs are a huge group of post-transcriptional regulators that control cellular and developmental processes by targeting messenger RNAs (mRNA). Many of them are significantly upregulated or downregulated in relation to different BC stages, increased local recurrence risk, and overall survival (Table 3).

Results of a single-miRNA prognostic power are confusing and, in many cases, contradictory. The panel consisting of multiple miRNA might enable a more precise prognostic model and efficient diagnostic tool [67,68]. The same principle was used by the authors Zhou et al. who, in a study conducted on two cohorts from The Cancer Genome Atlas (training, *n* = 596 and testing set, *n* = 319) identified miRNA expression profiles model consisting of 14miRNAs that could also serve as potential molecular targets of therapeutic strategies. This model was able to divide patients into high-score and low-score group with different overall survival and also proved the miRNA panel principle [61]. The expression of 71 miRNAs that correlates with prognostic significance was published by the authors Aure et al. [69]. Some of the miRNAs (miR-187, miR-210) may act as independent prognostic markers and their overexpression leads to more aggressive and invasive tumor phenotype [61,70,71]. Moreover, the study conducted on 68 patients with BC revealed the role of miR-9 asa promising predictor of local recurrence and lymph node metastases [72,73]. Also, the analysis of 139 BC tissue samples revealed that downregulation of miR-182-5p and miR-200b-3p represents independent prognostic parameter for disease recurrence in patients with luminal BC after endocrine therapy. Decreased disease-free survival is connected with the downregulation of miR-30 family (miR-30b-5p, miR-30c-5p) [62]. On the other hand, a global miRNA screen in primary tumors of 6 matched pairs of ER-positive, postmenopausal BC patients treated with tamoxifen (either recurrence-free or developed a recurrence) suggested that higher expression of miR-126 and miR-10a is seen in patients with longer relapse-free survival. Interestingly, the protective effect of miR-10a is suggested to be associated with the maintenance of the apoptotic capacity of cancer cells while miR-126 could be related to suppression of metastasis and reduction of tumor growth and proliferation [74].

Predictive tools in ER-positive BC like Mammaprint and Oncotype DX are unable to predict recurrence beyond five years [75]. Incorporating other molecular parameters could enhance the available predictive models. One study correlated miRNA expression and Oncotype DX recurrence score (RS). They detected reduced expression of let-7 family and high expression of miR-377-5p, miR-633b and miR-3648were associated with high RS scores [65]. On the contrary, let-7 family members are downregulated in tumors with high recurrence score [43]. What is interesting, not only upregulation or downregulation of miRNAs affect the process of carcinogenesis. Even the increase activity of selected miRNAs like miR-500a is connected with increased BC mortality [76].

There is a possibility to go deeper in the analysis of prognostic effect of particular miRNA. Single nucleotide polymorphisms can change the binding sites of miRNA for mRNA and alter the final effect. The study of Lee et al., conducted on 452 patients with EBC, provided evidence that the miR-196a rs11614913T>C polymorphisms are possible prognostic biomarkers for hormone receptor-positive BC. The patient with the CC genotype showed worse prognosis compared to TT or TC genotype [77].

In conclusion, various miRNAs are related to regulation of various mechanisms connected to carcinogenesis, such as processes of apoptosis, proliferation, tumor growth, or metastasis [74]. Therefore, the analysis of the miRNA expression, especially panels of multiple miRNAs, represents a promising tool enabling the evaluation of BC prognosis while its prognostic significance has been demonstrated by several authors [61,67,70].

## 5. miRNAs and Their Role in Endocrine Resistance

Almost 70% of BC cases are characterized by estrogen receptor (ER) positivity making these patients eligible for endocrine therapy including selective modulators of estrogen receptors (SERMs), selective estrogen receptor degrader (SERD) fulvestrant, and aromatase inhibitors (AIs). The collaborative meta-analysis of individual patient data with diagnosed EBC (*n* = 21,457) revealed that tamoxifen (TAM) reduces the mortality by 28% at 15-years follow-up and is recommended as a first line treatment for premenopausal BC patients. Moreover, PR measurement was not predictive in the respond to tamoxifen therapy. Therefore, the study was limited to ER-positive disease with 10,645 women [78]. The majority of luminal A BC responds well to endocrine therapy, but on the other side 40% of BC patients develop resistance [79]. This finding explains the fact that luminal A BC have relatively high rates of recurrence and metastases and are among the most threatening types of BC [80]. Endocrine resistance is either primary (when the patients relapse within the first 24 months of endocrine therapy) or secondary (relapse of the BC is diagnosed while the patient is on adjuvant endocrine therapy after 2 years of treatment or within 12 months after completing treatment [81].

Multiple studies have been published evaluating the exact molecular mechanisms of endocrine resistance. These molecular pathways include modification of ERα receptor expression, regulation of signal transduction pathways, altered expression of miRNAs, balance of regulatory proteins, and genetic polymorphisms [82]. In our systematic review, we focused on the already published studies regarding the connection of up/downregulation of miRNA and endocrine therapy resistance.

miRNAs can be both tumor suppressors and oncogenes depending on cellular system. Their expression inversely correlates with their targeted mRNAs. Selected miRNAs are consistently upregulated (miR-181-b) with functional targets including *HEY1*, *CA2*, *PIK3R1*, *LYN*, *ESR1*, *JUN*, *STAT1*, *MYB*, *BCL2*, *CYCS*, *BAMBI*, *CTGF*, and *SOX9* or downregulated (miR-342-3p/5p) with functional targets including *FYN*, *TGFBR1*, *COL4A6*, *CDKN1A*, and *Ephrins EPHA4/7* in all MCF-7/TAM-resistant cell lines. Furthermore, altered expression of miR-190b and miR-516a-5p in TAM-resistant cells revealed the predictive treatment outcome in a cohort of ER+ BC patients with tamoxifen mono-therapy [83]. Single miRNA prediction model of resistance has a low predictive power with different results across the studies. That is why the research is focused on multi miRNA expression profiles and predictive models [25]. Study of Joshi et al. showed alterations in expression of 131 different miRNAs in TAM-resistant cells [83]. What is more, Nam et al. identified undescribed network clusters consisting of miR-146a, miR-27a, miR-145, miR-21, miR-155, miR-15a, miR-125b, let-7s and miR 221/222, which contribute both to TAM and fulvestrant resistance. This “integrative network” focused on joint miRNA-mRNA expression profile, miRNA-target mRNA relationship, miRNA upstream regulators, and cancer context [84]. miR 221/222 cluster is one of the most important regulators of estrogen receptors and have a crucial role in TAM resistance [85,86]. Protein p27 representing a predictive factor for tamoxifen therapy response is one of the targets of miR 221/222. P27 is reduced by 28–50% in miRNA 221/222 overexpressing MCF-7 BC cells. Miller et al. demonstrated that the overexpressed miR-221/222 could be a potential pattern not only of TAM but also fulvestrant resistance. Moreover, the first mention of the relationship between miR-221/222 expression and HER2/neu overexpression was observed in primary BC samples resistant to TAM therapy [85,86,87].

miRNAs are also very promising novel therapeutic approach to sensitize and suppress the growth of TAM-resistant tumors [88]. Zhang et al.demonstrated downregulatedexpression of miR-135a in ERα+BC cells with acquired TAM resistance; however, the overexpression of miR-135a partially resensitized cells to TAM therapy through the activation of ERK1/2 and AKT pathways and miR-135a targeted genes *ESR1*, *ESRRA*, and *NCOA1* [89]. The same principle was seen in case of miR-27 that increases the levels of ERα [90]. miR-34 could also be an effective target to reverse TAM resistance. The first anti-cancer miRNA drug that mimics miR-34 has now reached first phase of clinical testing in patients withprimary liver cancer. miRNA-based therapeutics could have an important effect on tumor, because a single miRNA could affect multiple therapeutic targets with no side effects [91]. The other target could be miR873/CDK3 complex that also plays a crucial role in ERα signaling and TAM resistance. Norcanthraridin used as an anti-cancer drug in China sensitize resistant cells to TAM through the modulation of miR873 axis with downregulation of CDK3 [92,93]. The other potential therapeutic targets are represented by let-7 family [94], miR-155 [95], miR-192-5p, miR500a-3p and miR-206 [94,96,97]. Differently expressed miRNAs in tamoxifen-resistant and sensitive BC is summarized in Table 4 and Table 5.

miRNAs may play acritical role also in resistance to fulvestrant (Table 6) that is used as asecond-line therapy in cases of metastatic BC [120]. Acquired fulvestrant resistance is an ERα independent condition with alteration of growth hormone pathways, glycoprotein 88 overexpression [121], and functional methylation of the ER promoter region [120,122]. miR-143, miR-145, miR-137, miR-424, miR-21 may play important roles in fulvestrant resistance as well as miR-221/222, which is upregulated in fulvestrant-resistant cancer cell lines [123,124,125].

Aromatase inhibitor (AI) therapy is afirst line treatment in postmenopausal women. The study of Vilquinet al. showed for the first time that the deregulated miRNA expression of miR-23b, miR-484, miR-21, miR-301, and miR-193a activates the AKT pathway and causes the acquisition in AI therapy resistance. Therapeutic blocking of miRNA expression like miR-125b could initiate greater response to AI endocrine therapy [127]. Another pathway of AI resistance is the metabolic reprogramming by miR-155, where the overexpression of selected miRNA is correlated with poor response to AIs [128]. The summary of upregulated miRNAs in AI-resistant BC is shown in Table 7.

CDK4/6 inhibitors like Palbociclib, which are the newest therapy modality, could be used in combination with TAM in resistant BC. One of the markers that is able to indicate effective therapy by CDK inhibitors could be the overexpression of miR-18a, which is related to tumors with higher proliferation index and identifies high-risk luminal BC patients [103]. On the other hand, downregulation of miR-223 confers resistance to CDK 4/6 inhibitors in luminal BC and identifies aggressive DCIS as an early event during mammary carcinogenesis. miR-223 expression is increased in case of palbociclib therapy and is a marker of effective treatment [131].

As was mentioned above, the dysregulation of miRNAshas been considered as a critical mediator of cancer development and progression [89]. Although the anti-cancer drugs are initially effective, the clinical benefits from their use are limited by the development of endocrine resistance. An identification of target genes, protein-protein interactions of deregulated miRNAs and new knowledge about the resistance to anti-cancer drugs can give a base for the development of novel therapeutics targeting specific molecules [83,88,89].

## 6. Conclusions

Luminal A BC is the most common intrinsic type of malignant breast tumors. Despite their favorable prognosis and availability of targeted hormonal therapy, we still need to find new efficient prognostic and predictive markers for individualized approaches and personalized therapy. New studies emerging in the last decade show a significant heterogenicity in luminal A BC type [8]. Recent evidence suggests that epigenetic changes including the expression of various miRNAs can distinguish the tumors with worse overall prognosis and different answers to hormonal therapy including tamoxifen, fulvestrant, or aromatase inhibitors. Complex miRNA expression profilesare also a promising target for an advanced therapy of luminal A BC. The heterogeneous nature of malignant diseases requiresmore precise management of individual patients for the purpose of personalized treatment within the concept of precision medicine. The determination of specific levels of expression of different miRNAs or panels of multiple miRNAs enable the identification of valuable BC biomarkers. Recently, a rich evidence has been published on the specific levels of expression of individual miRNAs at settings of BC, which deeply contribute to the significance of the potential use the analysis of epigenetic changes in the management of BC. Despite promising results, the evaluation of the applicability of miRNA expression in the prediction and prognosis of BC has been associated with certain limitations, e.g., the number of enrolled samples and the requirement of larger samples [73]. Also, discrepancies between various studies regarding e.g., the role of specific miRNA evaluated by different authors may be associated with their specific focus, demonstrating that different disease settings and study designs yield different candidates [74]. Regarding the evaluation of the likelihood of recurrence and resistance to therapeutics such as endocrine resistance [83,88,89], the expression of miRNAs should be tested in primary tumor sites, ideally in multicenter studies. Moreover, in vitro evaluation might contribute to the functional characterization of specific miRNAs [62]. Above all, the role of miRNA expression in liquid biopsies highlights its role as a non-invasive biomarker [67]. Nevertheless, overcoming the limitations observed so far in the investigation of the applicability of miRNA expression or panels of multiple miRNAs in the diagnosis or determination of the strategy of BC treatment is necessary for a better understanding of the role of miRNA within the heterogenous nature of BC required for further progress in the usability of miRNAs in BC management. In conclusion, a personalized approach using the miRNA diagnostics and targeted therapy could lead to a substantial change in BC paradigm, exact estimation of patient prognosis, and overall survival.

## Figures and Tables

**Table 1:** The expression profiles of miRNAs in LumAearly breast cancer (EBC).

miRNA	Patients/Specimen Characteristics (Number of Patients/Specimens)	Results	Reference
miR-16, miR-21, miR-155, miR-195	Serum from patients with BC (*n* = 49) and healthy controls (*n* = 19)	Increased level in LumA EBC	[40]
miR-195	Blood from patients with BC (*n* = 83) (ductal type—71%, LumA epithelial subtype—63%, early stage—71%, in situ—12%) and healthy controls (*n*= 63)	Higher expression in EBC patients	[41]
miR-29a, miR-181a, miR-652	Blood from patients with a new diagnosis of LumA-like BC (*n* = 54) and healthy control participants (*n* = 56)	Reduced expression in LumA-like BC women	[42]
miR-23a-3p, miR-152-3p	Blood samples from patients with BC (*n* = 106) (LumA, *n* = 23) and healthy control (*n* = 96)	Lower level in patients with LumA	[29]
miR-338-3p, miR-223, miR-148a	Blood samples before and after surgery of post-menopausal patients with ER+and HER2-early stage of BC (*n* = 24)	Lower level in post-operative ER+ EBC post-menopausal women	[43]
miR-1273g-3p	MCF-7 BC cells; patients with BC (*n* = 39) and healthy controls (*n* = 40)	Increased expression in MCF-7 cells and BC patients	[44]

**Table 2:** miRNA pattern in LumA BCassociated with metastasis.

miRNA Pattern	Study Design/Model	Result	Ref.
↑ miR-331 ↓ miR-195	Metastasized BC LumA patients vs. patients with local disease or healthy controls		[45]
↑ miR-203	MCF-7	↓ estradiol-induced viability, migration and invasion ↓ ERα protein expression	[49]
↓ miR-6744-5p	Anoikis-resistant sub-cell line (MCF-7-AR6)		[50]
↑ miR-6744-5p	MCF-7	↑ anoikis sensitivity ↑ E-cadherin	[50]
↑ miR-765		↓ proliferation, migration, invasiveness	[53]
↑ miR-628		↑ migration, invasiveness ↑ E-cadherin ↓ vimentin, Snail	[54]
↓ miR-340		↑ migration, invasiveness	[55]
↓ miR-200c ↑ miR-30c	MCF-7-derived mammospheres	↑ stem cells markers (*OCT4*, *SOX2*, *c-MYC*) ↑ EMT-related genes (*SNAIL1*, *CDH2*, *TWIST1/2*)	[56]
↓ miR-145-3p	MCF-7 (metastasisinduced and cancer environment imitated)		[57]
↑ miR-520c-3p	MCF-7, T47D	↓ migration ↑ E-cadherin ↓ vimentin ↓ fibronectin	[58]
↑ miR-206		↓ migration, invasiveness, EMT ↓ TGF-β, NRP1, SMAD2	[59]
↓ miR-190	T47D		[60]

EMT, epithelial-mesenchymal transition, NRP1, neuropilin-1; TGF-β, transforming growth factor beta; Explanatory notes: ↑ increase, promotion; ↓ decrease, inhibition.

**Table 3:** miRNA associated with worse prognosis, local recurrence, and overall survival.

Upregulated miRNA	Reference	Downregulated miRNA	Reference
miR-187	[61]	miR-203	[49]
miR-210	[61]	miR-182-5p	[62]
miR-224	[61]	miR-200b-3p	[62]
miR-9	[61]	miR-30b-5p	[62]
miR-1266	[63]	miR-30c-5p	[62]
miR-128-3p	[64]	Let-7 family	[65]
miR-661	[64]	miR-891a-5p	[64]
miR-296-3p	[64]	miR-383-5p	[64]
miR-196a	[66]	miR-1295a	[64]

**Table 4:** Differently regulated miRNA in tamoxifen-resistant BC.

**Downregulated miRNA in Tamoxifen-Resistant BC**		
**miRNA**	**Target Gene**	**Reference**
miR-106b	*YWHAG, YWHAZ*	[83]
miR-125a-3p	*CDK3*	[93]
miR-135a	*FOXM1, ERK1/2, AKT1*	[83]
miR-186-3p	*EREG*	[98]
miR-26a	*E2F7*	[99]
miR-27b-3p	*NR5A2, CREB1*	[100]
miR-33b	*FYN*	[83]
miR-342-3p/5p	*FYN*	[83]
miR-378a-3p	*GOLT1A*	[101]
miR-449a	*ADAM22*	[88]
miR-491-5p	*YWHAG, YWHAZ*	[83]
miR-577	*YWHAG, YWHAZ*	[83]
miR-593	*SNAI2*	[83]
miR-873	*CDK3*	[92]
miR-942	*YWHAG, YWHAZ*	[83]
miR-96	*YWHAG, YWHAZ*	[83]
**Upregulated miRNA in Tamoxifen-Resistant BC**		
**miRNA**	**Target Gene**	**Reference**
miR-10b	*HDAC4*	[102]
miR-18a	*MYBL2*	[103]
miR-101	*MAGI2, Akt*	[91]
miR-155	*SOCS6-STAT3*	[95]
miR-181b	*STAT1,MYB, BCL2, SOX9*	[83,104]
miR-192-5p	*ERα*	[96]
miR-196a	*Hox, Fox, Cdkinhib.*	[66]
miR-21	*TIMP3, ADAM*	[105,106]
miR-221	*P27, ER* *ɑ*	[107]
miR-222	*P27, ER* *ɑ*	[107]
miR-335-5p/3p		[108]
miR-519a	*CDKN1*	[109]
miR-663b	*TP73*	[110]
miR-92a-3p		[87]

**Table 5:** Differently regulated miRNA in tamoxifen sensitive BC.

**Downregulated miRNA in Tamoxifen Sensitive BC**		
**miRNA**	**Target Gene**	**Reference**
miR-301	*PTEN, Akt*	[111]
**Upregulated miRNA in Tamoxifen Sensitive BC**		
**miRNA**	**Target Gene**	**Reference**
miR-148a	*ALCAM*	[112]
miR-152	*ALCAM*	[112]
miR-200b/c	*ZEB1*	[113,114]
miR-214	*UCP2*	[97]
miR-261	*AGR*	[115]
miR-27a	*ZBTB10*	[90]
miR-320a	*ARPP-19*	[93]
miR-34	*CCND1, CDK4/6*	[116,117,118]
miR-375	*MTDH, ZEB1, SNAI2*	[119]
miR-451	*HER, EGFR, MAPK*	[80]
miR-575	*AGR*	[115]

**Table 6:** Differently regulated miRNA in fulvestrant-resistant BC.

**Downregulated miRNA in Fulvestrant-Resistant BC**		
**miRNA**	**Target Gene**	**Reference**
miR-137	*SRC3*	[126]
miR-143		[126]
miR-145		[126]
miR-424	*PI3K/Akt/mTOR*	[126]
**Upregulated miRNA in Fulvestrant-Resistant BC**		
**miRNA**	**Target Gene**	**Reference**
miR-21	*PI3K/Akt/mTOR*	[126]
miR-221	*PCDH10*	[126]
miR-222	*CAMs KEGG pathway*	[126]

**Table 7:** Differently regulated miRNA in aromatase-resistant BC.

Upregulated miRNA in Aromatase Inhibitor-Resistant BC			
miRNA	Target Gene	Reference	**Aromatase inhibitor**
miR-125b	*Akt/mTOR*	[127]	Letrozole, anastrozole
miR-128a	*TGFbRI*	[129]	Letrozole
miR-155	*HK2, STAT3*	[128]	Anastrozole
miR-205	*Akt/mTOR*	[127]	Letrozole/anastrozole
miR-432-5p	*TGF-* *β*	[130]	Letrozole/anastrozole
miR-433-3p	*MAPK*	[130]	Letrozole/anastrozole
